# Frontal wrinkles in the elderly: prevalence, patterns and association of sleep lines with sleep quality^☆^

**DOI:** 10.1016/j.abd.2026.501454

**Published:** 2026-08-28

**Authors:** Jane Elizabeth Malheiros Souza de Campos, Ralph Vighi Da Rosa, Jéssica Trettin Puchalski, Natália Wirowski, Hiram Larangeira de Almeida

**Affiliations:** a Postgraduate Program in Health and Behavior, Universidade Católica de Pelotas, Pelotas, RS, Brazil; b Department of Dermatology, Universidade Católica de Pelotas, Pelotas, RS, Brazil

Dear Editor,

Skin aging is influenced by intrinsic factors, such as genetics and cell metabolism, as well as extrinsic factors, such as environmental exposures and lifestyle choices.^1^

Sleep wrinkles develop in response to the distortion created when the face is pressed against any sleeping surface. Compression, shear, and stress forces result in facial distortion during side- or prone-sleeping positions. In contrast, the only external forces acting on the face in the supine position are gravitational ones.^2^

In a youthful face, wrinkling associated with facial expressions is transient, and sleep wrinkles disappear upon waking. The extent and severity of wrinkling vary with skin aging, in response to intrinsic and extrinsic influences combined with repetitive force patterns.^2^ Sleep wrinkles differ from expression lines in their mechanism of origin (external forces vs. internal muscle contraction), location, and directionality (they are generally perpendicular to one another).^2^

Few studies have specifically examined the effect of sleep deprivation on skin function, but it has been hypothesized that lack of sleep and psychological stress could impair skin integrity.^3^, ^4^, ^5^

The aim of this study was to evaluate the prevalence and pattern of dynamic wrinkles (glabellar and frontal) and sleep wrinkles in an elderly population in the city of Pelotas, RS, and to assess the potential relationship between the latter and sleep quality.

This is a cross-sectional study involving patients aged 60 years or older treated in 2025 at the Geriatrics outpatient clinic of Universidade Federal de Pelotas. Patients with dementia, those who had undergone cosmetic surgery, and those who regularly used botulinum toxin were excluded from the study. This study was submitted to the Research Ethics Committee (CEP) of Universidade Católica de Pelotas and approved under opinion number 7,294,061.

A questionnaire collected data on the following variables: sex, skin color, education level, income, and history of aesthetic procedures. Sleep quality in the elderly was assessed using the Pittsburgh Sleep Quality Index (PSQI),^6^ an instrument that evaluates sleep quality over the 30 days prior to the questionnaire's administration, classifying patients as having “good sleep quality” (PSQI ≤ 5) or “poor sleep quality” (PSQI > 5).

Participant photographs were taken during the medical consultation. Images of the frontal and lateral facial regions were recorded—with a minimum of six photos per patient—and evaluated by two dermatologists to ensure consensus. Frontal wrinkles were quantified based on up to four and more than five horizontal lines, a criterion selected by the authors. Glabellar wrinkles were quantified based on vertical lines and the presence or absence of horizontal lines. Sleep lines were quantified and their laterality identified (as illustrated in Fig. 1, Fig. 2).

**Fig. 1:**
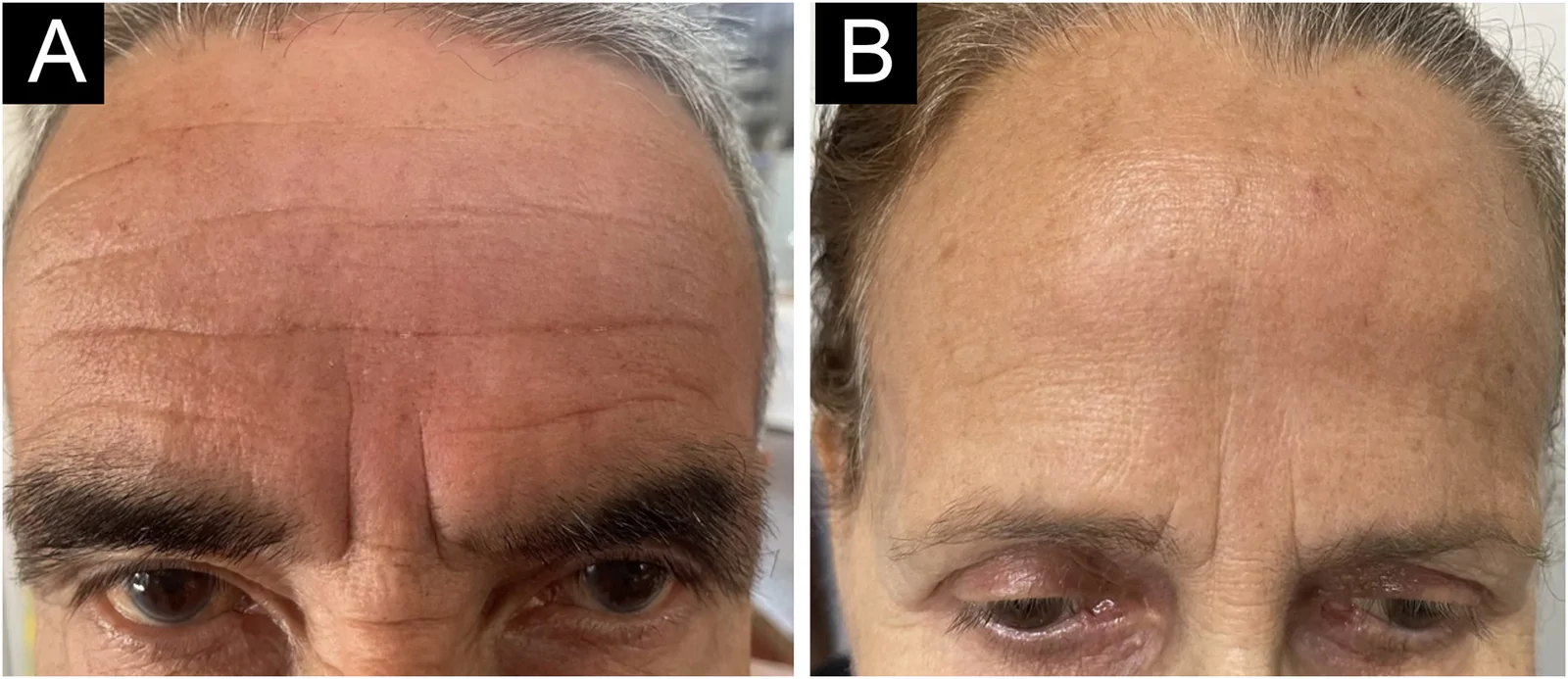
(A) Most common glabellar wrinkle pattern: two vertical lines with a horizontal wrinkle; the patient also has four well-defined forehead wrinkles. (B) Second most common pattern: three vertical lines with a horizontal wrinkle.

**Fig. 2:**
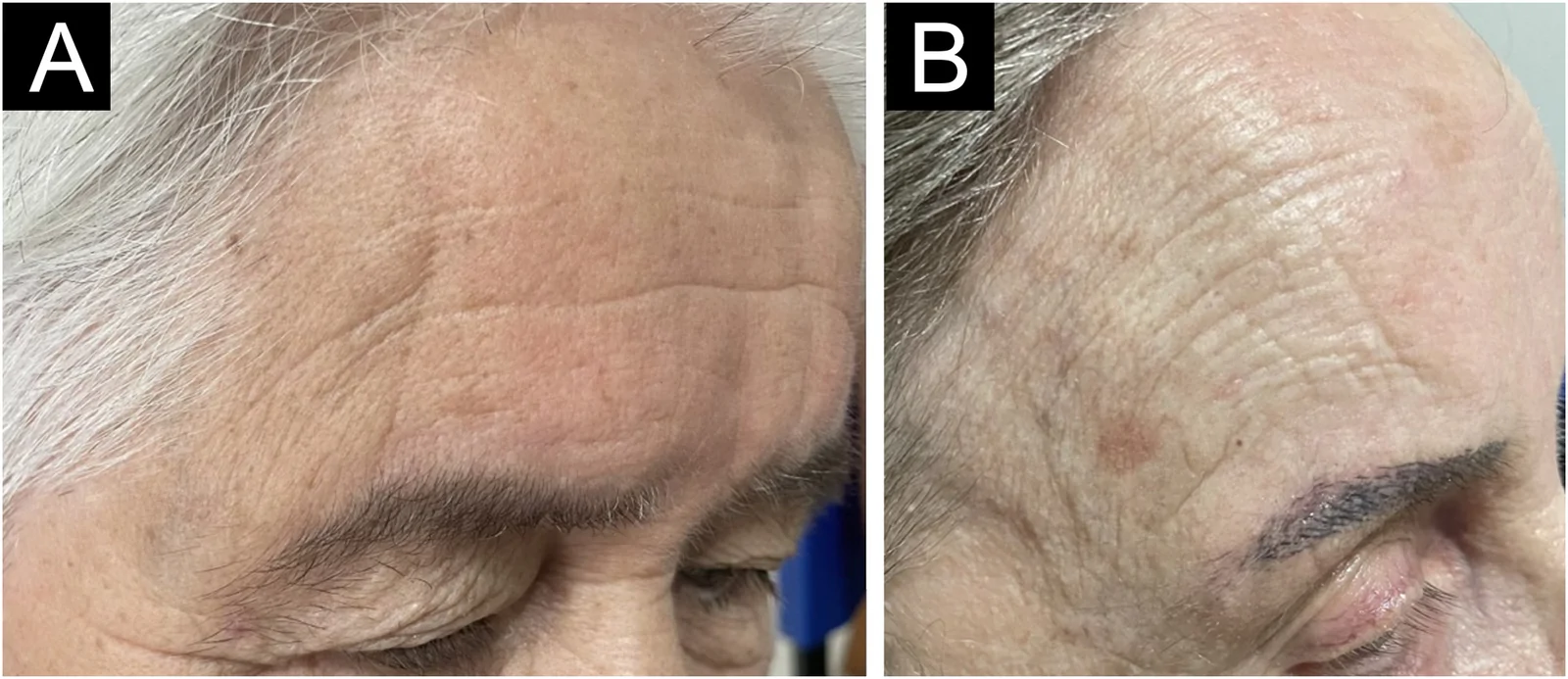
(A) A sleep wrinkle on the right. (B) Two sleep wrinkles on the right.

Statistical analysis was performed using IBM SPSS Statistics software, version 31.0.2.0 (free version). Scores obtained from psychometric scales were used for association analyses with the participants' wrinkles. In cases of normal distribution (p > 0.05), data were expressed as mean and standard deviation.

The total sample size consisted of 201 participants, of which 75.6% (n = 152) were women. The participants’ mean age was 75.04 ± 6.93 years. Regarding skin color, 79.6% (n = 160) of the individuals were classified as white. In terms of educational level, 62.7% (n = 126) of the participants had up to five years of schooling. Regarding socioeconomic classification, 91% (n = 183) fell into classes D and E.

Good sleep quality was found in 27% (n = 54) of the individuals, while 73% (n = 146) exhibited poor sleep quality. Forehead wrinkles were found in 183 (91.5%) of those examined, glabellar wrinkles in 197 (98.5%), and sleep wrinkles in 92 (46.0%) individuals.

Among glabellar wrinkles, the most prevalent morphology was 2+H (52%)—that is, two vertical wrinkles plus one horizontal wrinkle (Fig. 3A). Among forehead wrinkles, the most prevalent morphology was “multiple” in 36.5% of those examined, defined as five or more forehead wrinkles (Fig. 3B).

**Fig. 3:**
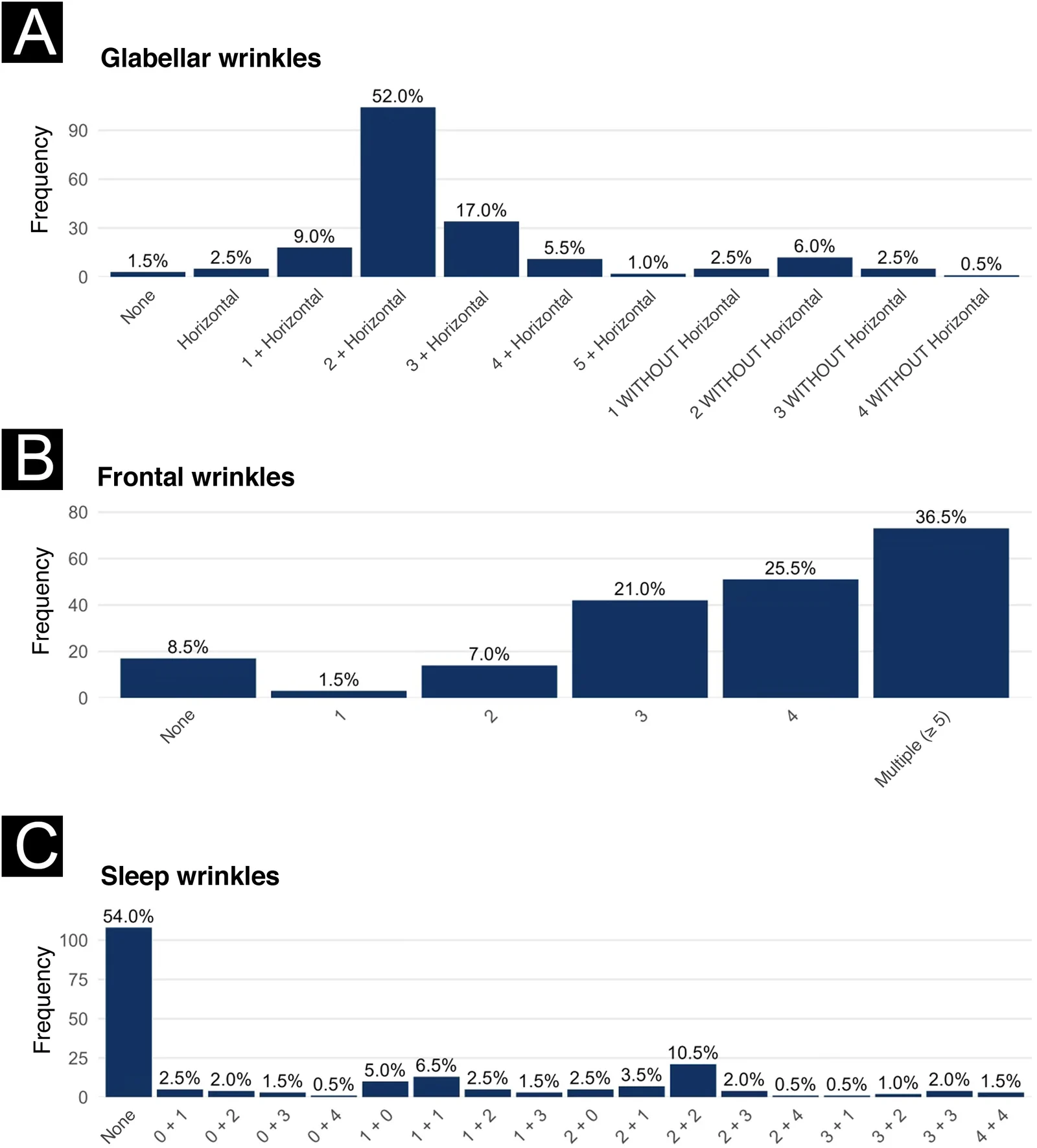
Wrinkle pattern – (A) Glabella – the most frequent pattern was two vertical wrinkles with a horizontal component (52%), followed by three vertical wrinkles with a horizontal wrinkle (17%). (B) Forehead – the majority of patients had more than five horizontal forehead wrinkles (36.5%). (C) Sleep wrinkles – the most common pattern was two wrinkles on each side (10.5%), followed by one on each side (6.5%).

Finally, among sleep wrinkles, the most prevalent morphology was 2 + 2 (10.5%)—that is, two sleep wrinkles on the right and two on the left (Fig. 3C).

In the analysis of the association between sleep wrinkles and sleep quality in the elderly, a dichotomous classification (present vs. absent) was used for wrinkles as well as for sleep quality (good vs. poor). Poor sleep quality was observed in 59 (64.1%) individuals with sleep wrinkles and in 68 (63.0%) individuals without them (Table 1); these prevalence rates were similar, with no statistically significant association (p > 0.05).

**Table 1:** Relationship between sleep wrinkles and sleep quality in the elderly individuals examined.

	Sleep wrinkles^a^ (Presence/Absence)		*n* (%)	p-value
	No	Yes		
Good sleep quality (PSQI ≤ 5)	40 (37.0)	33 (35.9)	73 (36.5)	0.864
Poor sleep quality (PSQI > 5)	68 (63.0)	59 (64.1)	127 (63.5)	
Total	108 (54.0)	92 (46.0)	200 (100.0)	

a Chi-square – variable with one missing.

Data on sleep wrinkles in the literature are scarce. When comparing the present study with existing literature, one study found an 11% prevalence of sleep wrinkles in a population of 542 female participants, as well as significant associations between the presence of sleep wrinkles and chronological age.^7^ However, that study analyzed only women aged 44 to 70, differing from the present research.

Another study, published in 2015, aimed to evaluate the effect of chronic poor sleep quality on indicators of skin health and aging.^3^ Sleep quality was also assessed using the Pittsburgh Sleep Quality Index (PSQI), and signs of skin aging were evaluated using a tool called SCINEXA (SCore for INtrinsic and EXtrinsic skin Aging), which comprises five items indicative of intrinsic aging and 18 items highly characteristic of extrinsic aging, in addition to an assessment of transepidermal water loss. The sample consisted of 60 exclusively female participants, with a mean age of 37.5 (±5.5) years in the “good sleepers” group and 39.6 (±5) years in the “poor sleepers” group. The analysis results indicated that poor sleepers exhibited higher values ​​for uneven pigmentation, fine lines, skin sagging, subcutaneous fat loss, and benign skin growths, suggesting that chronic poor sleep quality is associated with accelerated intrinsic aging.^3^

A relevant point for discussion is that, although our initial sleeping position is a conscious choice, we shift positions throughout the night as our bodies unconsciously attempt to minimize discomfort. With age, the frequency of position changes decreases from 27 to 16 per night, averaging 20 changes per night. Consequently, the time spent in each position increases with age. Side-sleeping is the most common position across studies—averaging 65% side, 30% supine, and 5% prone^2^ —yet it is uncommon for an individual to spend 100% of their sleep time in a single position.^2^ As skin loses elasticity, these lines progress and become permanent due to repetition and aging.^2^

Thus, consistent with existing literature, a high prevalence of sleep wrinkles was found in the studied population, although there was no association with sleep quality.

A relevant and positive aspect of this research was that the sample consisted exclusively of older adults (individuals aged 60 or older)—with a mean age of 75.04 (±6.93) years—recruited from a geriatric outpatient clinic. Furthermore, the sample consisted primarily of older women who were not in the perimenopausal stage. Consequently, the association between poor sleep quality and the menopausal transition—well-established in the literature—did not act as a confounding factor.^8^, ^9^, ^10^

One limitation of this study was that it assessed only wrinkles in the participants' frontal region, whereas sleep wrinkles can also appear on the cheeks, chin, nose, and under the eyes.

In conclusion, this study identified a high prevalence of frontal and glabellar wrinkles (91.5% and 98.5%) in patients who do not undergo aesthetic procedures, highlighting the importance of using botulinum toxin to prevent this type of wrinkle. There was no correlation between sleep wrinkles and sleep quality.

## Authors’ contributions

Jane Elizabeth Malheiros Souza de Campos: Approval of the final version of the manuscript; design and planning of the study; drafting and editing of the manuscript; collection, analysis, and interpretation of data; critical review of the literature; critical review of the manuscript.

Ralph Vighi Da Rosa: Approval of the final version of the manuscript, design and planning of the study, drafting and editing of the manuscript, collection, analysis, and interpretation of data; effective participation in research orientation, critical review of the literature, and critical review of the manuscript.

Jéssica Puchalski Trettim: Approval of the final version of the manuscript, design and planning of the study, drafting and editing of the manuscript, collection, analysis, and interpretation of data, effective participation in research orientation, critical review of the literature, and critical review of the manuscript.

Natália Wirowski: Approval of the final version of the manuscript; design and planning of the study; drafting and editing of the manuscript; collection, analysis, and interpretation of data; critical review of the literature; critical review of the manuscript.

Hiram Larangeira de Almeida Jr.: Approval of the final version of the manuscript, design and planning of the study, drafting and editing of the manuscript; collection, analysis and interpretation of data; effective participation in research orientation, critical review of the literature, and critical review of the manuscript.

## Financial support

None declared.

## Research data availability

The entire dataset supporting the results of this study was published in this article.

## Conflicts of interest

None declared.

## References

[1] Hussein R.S., Bin Dayel S., Abahussein O., El-Sherbiny A.A. (2025). Influences on skin and intrinsic aging: biological, environmental, and therapeutic insights. J Cosmet Dermatol.

[2] Anson G., Kane M.A., Lambros V. (2016). Sleep wrinkles: facial aging and facial distortion during sleep. Aesthet Surg J.

[3] Oyetakin-White P., Suggs A., Koo B., Matsui M.S., Yarosh D., Cooper K.D. (2015). Does poor sleep quality affect skin aging?. Clin Exp Dermatol.

[4] Poljsak B., Godic A., Starc A., Dahmane R. (2016). The neglected importance of sleep on the formation and aggravation of facial wrinkles and their prevention. J Cosm Dermatol Scie Appl.

[5] Xerfan E.M.S., Facina A.S., Tomimori J., Tufik S., Andersen M.L. (2022). Sleep lines on the skin: an overview of possible causes beyond a purely mechanical etiology. Int J Dermatol.

[6] Bertolazi A.N., Fagondes S.C., Hoff L.S., Dartora E.G., Miozzo I.C., de Barba M.E. (2011). Validation of the Brazilian Portuguese version of the Pittsburgh sleep quality index. Sleep Med.

[7] Jdid R., Ezzedine K., Latreille J., Galan P., Hercberg S., Malvy D. (2014). MC1R major variants are a risk factor of sleep lines in Caucasian women. J Eur Acad Dermatol Venereol.

[8] Shaver J.L., Woods N.F. (2015). Sleep and menopause: a narrative review. Menopause.

[9] Xerfan E.M.S., Sartor A., Samama M., Facina A.S., Tomimori J., Andersen M.L. (2022). Reproduction, skin aging, and sleep in middle-aged women. Clin Dermatol.

[10] Jang S.I., Lee M., Han J., Kim J., Kim A.R., An J.S. (2020). A study of skin characteristics with long-term sleep restriction in Korean women in their 40s. Skin Res Technol.

